# Mechanism of action and efficacy of LY2109761, a TGF-β receptor inhibitor, targeting tumor microenvironment in liver cancer after TACE

**DOI:** 10.18632/oncotarget.23193

**Published:** 2017-12-11

**Authors:** Xiaojun He, Xiaopeng Guo, Hongsen Zhang, Xiangchuang Kong, Fan Yang, Chuansheng Zheng

**Affiliations:** ^1^ Department of Radiology, Union Hospital, Tongji Medical College, Huazhong University of Science and Technology, Wuhan, China; ^2^ Department of Radiology, Wuhan Children's Hospital (Wuhan Maternal and Child Healthcare Hospital), Tongji Medical College, Huazhong University of Science and Technology, Wuhan, China

**Keywords:** tumor growth rate (TGR), smad, transcatheter arterial chemoembolization (TACE), LY2109761, hepatocellular carcinoma ( HCC)

## Abstract

TACE (transcatheter arterial chemoembolization) has been recognized as an effective palliative treatment option for patients with HCC, however, the medium-long term efficacy of it remains modest. LY2109761, a TGF-β receptor inhibitor, was confirmed to reduce tumor cell growth, intravasation, and metastatic dissemination of HCC cells through different molecular mechanisms. This study aims to investigate the treatment effect of combining TACE therapy with LY2109761- a TGF-β receptor I kinase inhibitor on suppressing tumor growth and metastasis in a rabbit VX2 tumor model. The molecular mechanisms underlying the biological activities of LY2109761 was also evaluated through an *in vitro* model. And we found that LY2109761 could inhibit cell proliferation by down-regulating the phosphorylation of Smad-2 as well as improved the therapeutic effect of TACE in a VX2 hepatocellular carcinoma model. And we further found that LY2109761 may play a modulating role in the process of T cell transformation. Hence, based on those obsevations in our research, we concluded that combing LY2109761 with TACE for the treatment of VX2 rabbit liver cancer can help inhibit tumor growth as well as increase the tumor cell necrosis after TACE.

## INTRODUCTION

Hepatocellular carcinoma (HCC) is one of the most common tumors, accounting for an estimated 700000 deaths annually worldwide [1]. Up to now, HCC has become a major health problem worldwide owing to its high mortality, morbidity, and an increasing incidence [2, 3]. Due to the hidden nature of onset of HCC, most patients are already at the advanced stage at the time of diagnosis and are not candidates for curative treatment like surgical resection and liver transplantation [4–6]. As it is impossible to perform such therapies, patients with advanced HCC have to rely on non-surgical therapies such as chemotherapy, transcatheter arterial chemoembolization or embolization (TACE or TAE), radiotherapy, targeted therapy, to prolong their survival time [7–10]. Transarterial chemoembolization(TACE), recognized as an effective palliative treatment option for patients with advanced HCC, is now widely performed for unresectable liver cancer which classified as intermediate stage according to the Barcelona Clinic Liver Cancer (BCLC) staging system [11–13]. A large body of evidence supports that TACE is not only effective for HCC of intermediate stage but also effective for early and advanced HCC [6, 13–16]. However, in many cases tumor cells can adapt to the highly anaerobic microenvironment resulted from TACE through the negative feed-back response and incomplete embolization, which often correlated with the recurrence and metastasis of tumor [17, 18].

During the past decades, with a better understanding of tumor pathogenesis, some molecular targeted agents had began to emerge. Moreover, in the new era of the war against cancer, molecular targeted therapy based on molecular targeted agents(MTAs) had gradually became an indispensable component of the treatment regimen for patients with HCC [18]. However, low efficacy and drug resistance hamper the clinical application of MTAs, especially in the treatment of HCC. Most recently, more and more studies have focused on the combination of TACE with molecular targeted therapy in order to improve prognosis as well as reduce adverse reactions(ADRs). Although in many cases, such combination didn’t show significant improvement in term of overall survival, the combined therapy may provide new strategies for the treatment options of HCC. Nevertheless, previous treatments of HCC which merely focused on targeting the tumor cells doesn’t attain optimal therapeutic effect.

Recently, a growing body of literatures have highlight the fundamental role of tumor microenvironment in the processes of hepatocarcinogenesis, tumor invasion and metastasis. And biological agents that target components of the tumor microenvironment may provide an alternative to traditional tumor cell-directed therapy. Transforming growth factor (TGF)–b superfamily, as an important component of tumor microenvironment, plays a critical role in modulating the biological behavior of HCC. It is also considered as a hallmark of HCC cause an increased level of it in HCC patients often related with tumor progression and poor survival [19–21].

In view of the dual role of TGF-b in HCC pathogenesis, targeting the aberrant pathway of TGF has been a new research hotspot in the treatment of HCC. Recently, LY2109761, a TGF-b receptor inhibitor, was increasingly confirmed to serve as suppressor of the synthesis and release of connective tissue growth factor in tumor environment, which in turn reduced tumor cell growth, intravasation, and metastatic dissemination of HCC cells through different molecular mechanisms [22, 23].

Based on those observations, we deduced that combing TACE therapy with LY2109761 may play a role in modulating the behaviors of particular factors or cells existed in t anaerobic tumor microenvironment after embolization, which may suppress the proliferation and migration of tumor cells. So hence, in this study, we for the first time combined TACE therapy with LY2109761 to evaluate the treatment effect of this combined therapy on suppressing tumor growth and metastasis in a rabbit VX_2_ tumor model. And the molecular mechanisms underlying the biological activities of LY2109761 was investigated *in vitro* as well.

## RESULTS AND DISCUSSION

Previous researches have shown that a status of hypoxia, nutritional deficiency and interstitial fluid pressure reduction, Ph decrease, stellate cell activation and fibrosis was existed in liver cancer after embolization treatment [24–27]. Such changes in the biological properties of tumor cells in turn lead to changes in the microenvironment of the tumor. Hence, to achieve a better understanding of the alteration of tumor microenvironment before and after TACE in our study, we have evaluated the expression of TGF-β, E-cadherin, MMP9 and related factors in Group I and Group NS through the method of immunohistochemistry staining. The tumor and surrounding tissues were taken 3 days after TACE procedure. And all the stained tissues in slides were analyzed through light microscope and quantified by the H-score method. And to intensively investigate the inhibitory mechanisms and effect of LY2109761, we have treated two HCC cell lines (Hep3B and HepG2) with this agent *in vitro* model. Related expression of interest proteins and factors were detected and analyzed as well. Furthermore, we have intensively evaluated the mechanisms and efficacy of LY2109761 on targeting tumor microenvironment in liver cancer after embolization in a rabbit VX2 liver cancer model.

### An overexpression of TGF-β was present in the tumor microenvironment in hepatocellular carcinoma

**Figure 1:**
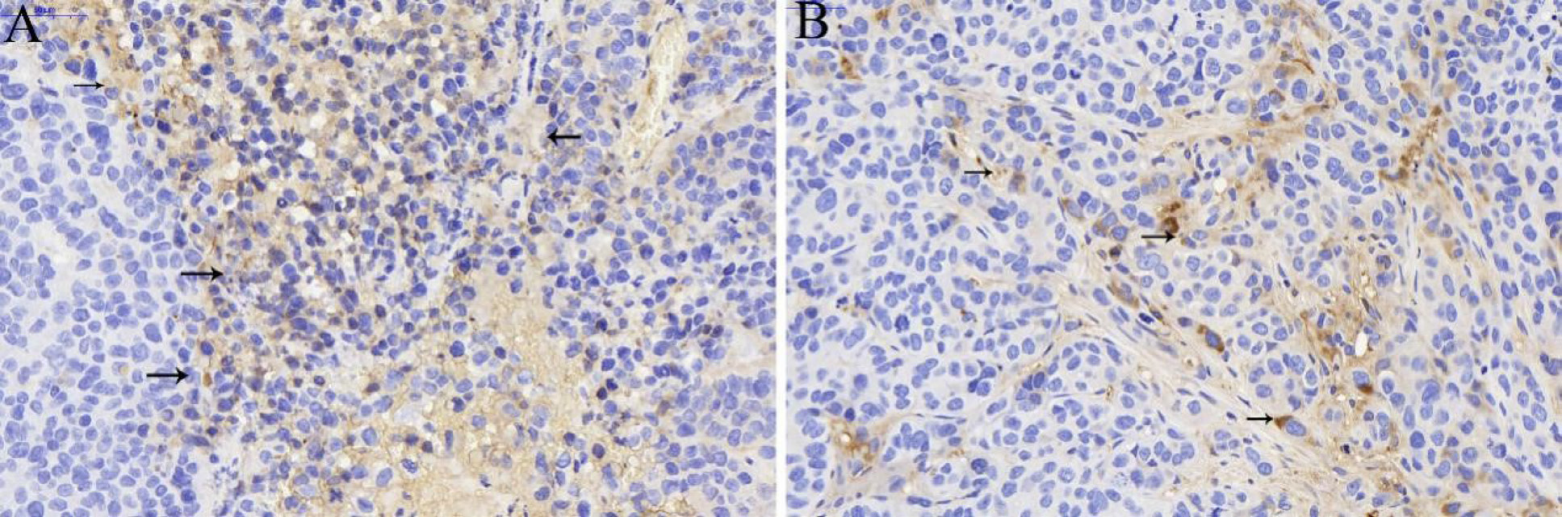
The expression of TGF-β (black arrow) in tumor stroma of Group NS (**A**) and Group I (**B**) a strong positive staining was shown in Group NS (**A**), whereas the degree of the staining of TGF-β on Group I (**B**) was much lower compared with Group NS.

TGF-β is considered as an intriguing pleiotropic factor in cancer because of its dual function as a tumor suppressor and promoter [28, 29]. Some researches have shown that TGF-β promotes cell migration, invasion, and anchorage-dependent growth in an experimental and *in vivo* preclinical model [22, 23]. Recently, a growing body of evidence have highlight the effect of TGF-β on promoting tumor growth and metastasis on liver cancer. Previous literatures have confirmed that TGF-β can be considered as a hallmark of HCC because an overexpression of it may tightly correlated with tumor progression and survival [19–21]. Therefore, in order to get a better understanding of the expression of TGF-β in rabbit liver cancer model, we have evaluated the expression of TGF-β before and after TACE treatment by immunohistochemistry. And finally the microscopic images of immunohistochemistry staining showed that other than surrounding tissues, the immunostaining of TGF-β in tumor stroma of hcc cells was strongly positive in Group NS (Figure 1A), whereas the immunostaining of TGF-β on Group I (Figure 1B) was much lighter compared with Group NS, which suggested that a lower expression of TGF-β was observed in rabbit liver cancer tissues after recieved TACE treatment.

Embolization with iodized oil for the treatment of liver cancer can on one hand blocks the blood supply of tumor cells as well as the stroma cells. On the other hand, iodized oil can also display as an cell killers due to the nature of it. However, in practical clinical work, conventional TACE can’t obtain a thorough necrocytosis of tumor cells. In addition, such condition would induce a hypoxia environment which in turn stimulates the growth and metastasis of the remaining tumor cells. As a consequence, in such circumstances, TGF-β pathways would play its role on inducing malignant biological behaviors as some researches confirmed before. Hence, targeting the TGF-β pathways may be a promising treatment strategy for HCC. In this regard, treatment strategies which combined Ctace with TGF-β pathway inhibitor may provide another interesting alternative for the treatment of HCC.

### LY2109761 inhibits cell proliferation by down-regulation the phosphorylation of Smad-2 and improved the therapeutic effect of TACE in a VX2 hepatocellular carcinoma model (*in vivo*)

The role of transforming growth factor-β1 (TGF-β1) in the progression of chronic liver damage, in which it stimulates fibrogenesis through activation of hepatic stellate cells, and the subsequent progression toward HCC is widely recognized [30–32]. In HCC, circulating TGF-β1 levels have been reported to be produced by the tumoral mass so that after surgery their concentration drops [33, 34]. Furthermore, plasma levels of TGF-β1 correlate with a more aggressive growth and spread having a higher vascular density and consequently a worst prognosis and shorter survival [35, 36]. Therefore TGF-β1 is commonly recognized as a hallmark of HCC and one of the most important pathways to be targeted. Some literatures have confirmed that LY2109761, a TGF-βRI kinase inhibitor could reduce HCC tumor growth by decreasing VEGF and connective tissue growth factor (CTGF) production, which lead to a lower vascularity and an interruption of the cross-talk between HCC and the surrounding stroma.

**Figure 2:**
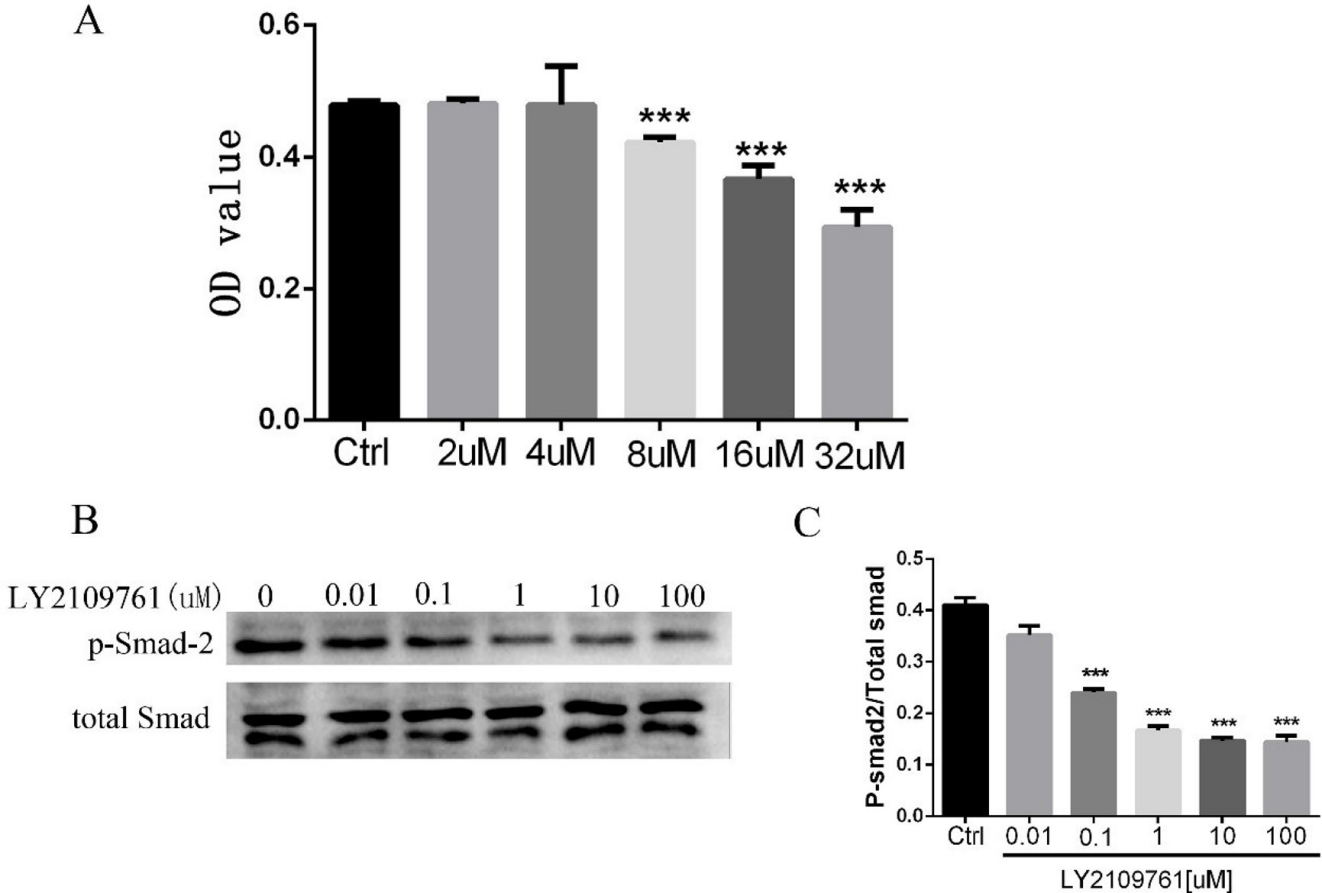
LY2109761 inhibits cell proliferation by down-regulation the phosphorylation of Smad-2 (**A**) The toxicity of LY2109761 on HepG2 cells was in a dose-dependent manner ranging from 8 uM -32 uM. (**B**) The expression of p-Smad-2 was down regulated by LY2109761 at the dose ranging from 0.1 to 100 Um. (**C**) The histogram revealed that the expression of p-Smad-2 was significantly decreased at the dose ranging from 0.1 to 100 uM/L.

In the present study, to evaluate the toxicity of LY2109761 on HCC cells, an CCK8 assay was performed. HepG2 cells were treated with LY2109761 at different concentrations followed by an 4h incubation in the CCK8 assay. And the result revealed that the toxicity of LY2109761 was in a dose-dependent manner (Figure 2A), ranging from 2 to 32 uM. LY2109761 was not cytotoxic at 2, 4 uM, whereas was cytotoxic at 8, 16, 32 uM. A growing body of evidence have highlights that the TGF-β/Smad pathway was tightly associated with the proliferation of tumor cells [23, 37]. Thus, we further investigated the expression of the phosphorylation of Smad-2 by the Western blot assay. And the data which consistent with the CCK8 assay, suggested that LY2109761 could down-regulate the expression of p-Smad-2 at a dose dependent manner, ranging from 0.1 to 100 uM (Figure 2B and 2C).

Based on those observations we founded above, in this study, we further evaluated the therapeutic effect of LY2109761 in an animal model and *in vivo* experiment, we combined TACE therapy with LY2109761 for the treatment of a rabbit VX2 liver cancer model to evaluate the therapeutic effect of such combined therapy. And the mechanisms underlying those effects were investigated as well.

**Figure 3:**
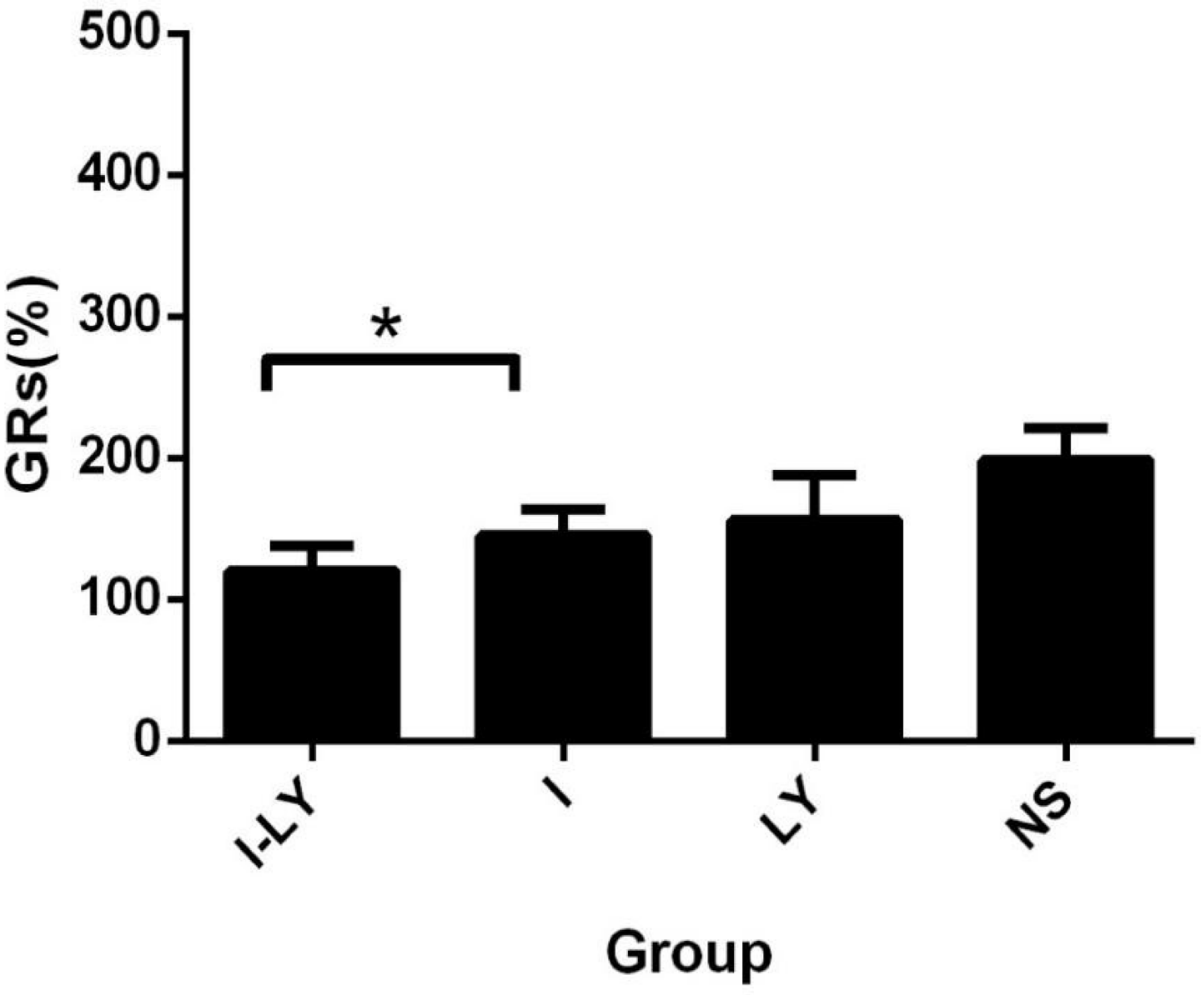
The GRs of tumors The tumor growth rate in the rabbits which undertaken the treatment of Group I-LY was much lower than Group I (*p* = 0.022 < 0.05).

All the experimental rabbits were randomly divided into four groups as described above and each group included 10 rabbits. The tumor sizes of rabbits of each group before and after TACE treatment were measured by enhanced CT and were summarized in Table 1. The tumor growth rates of each group were also included in Table 1. The One-Way ANOVA revealed that the GRs of the Group I-LY was significantly decreased compared with other groups (P value = 0.02 ＜ 0.05). And the analysis between Group I-LY and Group I also shared a promising significance. (*p* = 0.022 < 0.05). From the histogram (Figure 3), we concluded that other than simple lipiodol embolization, combining TACE therapy with LY2109761 for the treatment of rabbit VX2 liver cancer model yielded a better inhibitory effect on tumor growth after TACE.

**Table 1:** Tumor sizes of each group before and after TACE procedure and the tumor growth rate of each group

Groups	Tumor Size		Tumor Growth Rate (%) V_2_/V_1_ × 100%
	V1(one day before TACE procedure)	V2(seven days after TACE procedure)	
Group I-LY	1.805 ± 0.277	2.184 ± 0.391	116.59 ± 18.03^***^
Group I	1.894 ± 0.332	2.729 ± 0.489	145.17 ± 18.68
Group LY	1.812 ± 0.276	2.842 ± 0.320	156.24 ± 31.46
Group NS	1.806 ± 0.352	3.535 ± 0.486	198.68 ± 22.18
F value			19.989
P value			0.00

The tumor growth rate(TGR) of Group I-LY was significantly decreased compared with the other three groups (*p* = 0.00 < 0.05).

**Figure 4:**
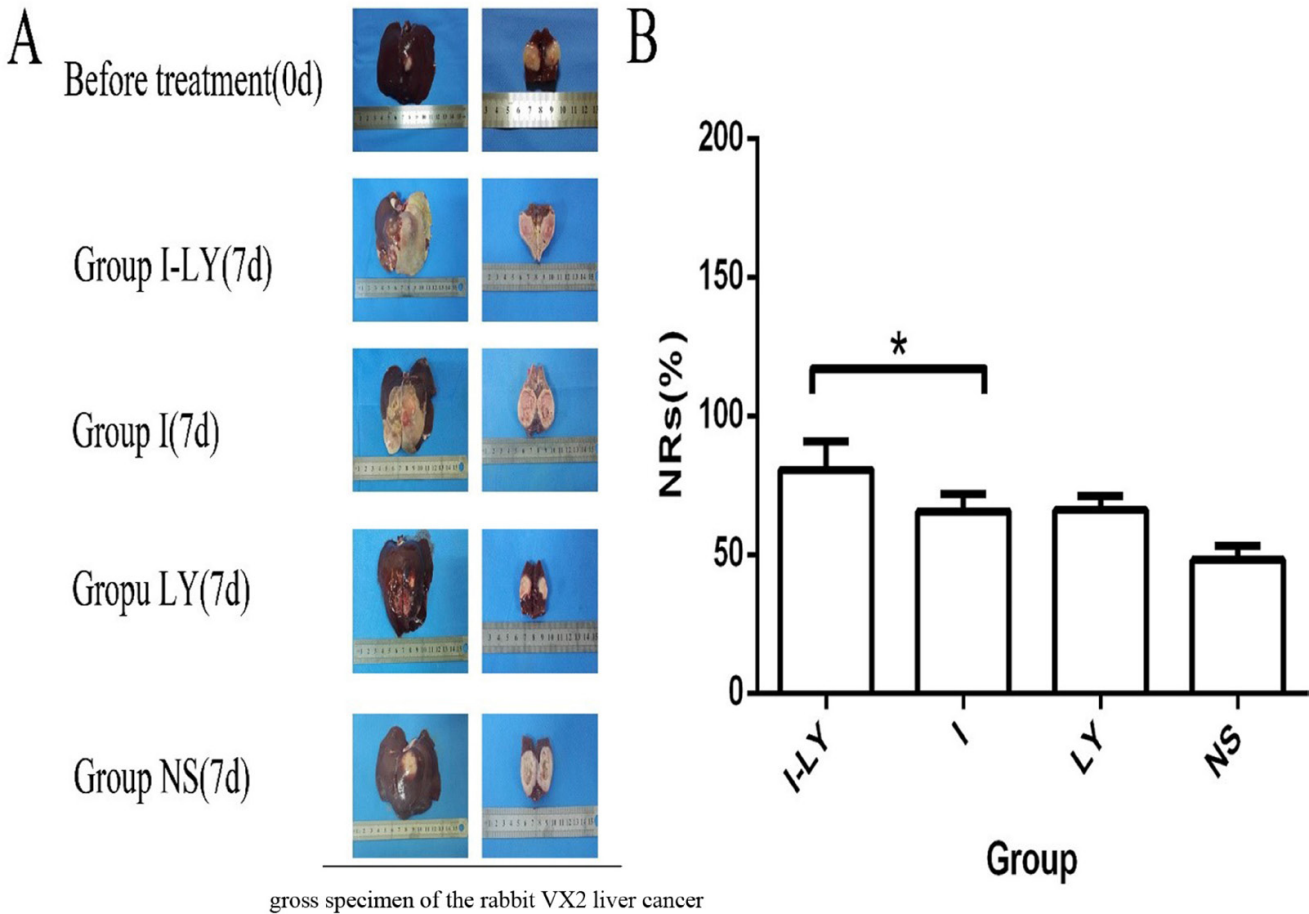
The NRs of tumors (**A**) The gross specimen of the rabbit VX2 liver cancer in different Groups. And the first picture means the holistic view of the tumor tissues, and the second picture means the longitudinal section of the tumor. (**B**) The necrosis rate in the four groups, and tumor tissues in Group I-LY aquired a much thorough necrosis compared with Group I (*p* = 0.03 < 0.05).

The necrosis rates of the four groups were also evaluated 7days after TACE treatment. The tumor samples were taken 7days after TACE treatment and were cut from the center where the necrosis area was measured. The necrosis area was calculated as S_1_=c_1_×d_1_, where c_1_ refered as the major axis of the necrosis area, and d_1_ refered to the minor axis of the necrosis area. And the area of the tumor section was calculated as S_2_=c2×d2, where c_2_ refered as the major axis of the tumor section , and d_2_ refered to the minor axis of the tumor section. And the necrosis rate was calculated as“NRs= S_1_/S_2_×100%. And our data revealed that embolization with LY2109761 obtained a more thorough necrosis of the tumor compared with simple lipdiol embolization (Figure 4A). And the data showed that the necrosis rates of liver cancer in Group I-LY, Group I, and Group LY were significantly increased compared with Group NS. And statistical analysis revealed that TACE combined with LY2109761 could significantly increase the necrosis rate of tumor cells compared with lipiodol embolization(*p* = 0.03 < 0.05) (Figure 4B).

Based on those observations, we concluded that LY2109761 combined with TACE treatment could not only inhibit tumor growth after TACE but also enhanced the anti-tumor effect of TACE, and in this regard, combining TACE with TGF-β inhibitor may thus yielding a promising therapeutic effect of HCC.

### LY2109761 suppressed cell migration and invasion of HCC cells as well as promoted the expressing of E-cadherin

In the last decade, transforming growth factor-beta 1(TGF-β1) has been recognized as a key driver in liver fibrosis, resulting in a higher risk of HCC development [38]. TGF-β1 has a double-sword role in many cancers including HCC: as an onco-suppressor it inhibits cell proliferation, whereas as a tumor promoter it triggers the epithelial–mesenchymal transition, which facilitates tumor spread and metastasis because of a downregulation of E-cadherin. The mechanisms regulating the switch from onco-suppressor to tumor promoter are still debated. However, in *in vitro* and *in vivo* models two distinct responses to TGF-β1 have been demonstrated; the ‘early’ response is associated with an epithelial, and the ‘late’ response with a mesenchymal, phenotype. These responses are correlated with a better or worse prognosis for HCC patients, respectively. The small molecule kinase inhibitor galunisertib selectively blocks TGF-β receptor 1 (TGF-βR1), thereby inhibiting the induction of the canonical Smad-2 signaling pathway. Smad-2 inhibition increases the E-cadherin level, reducing the migratory and invasive capabilities of HCC.

**Figure 5:**
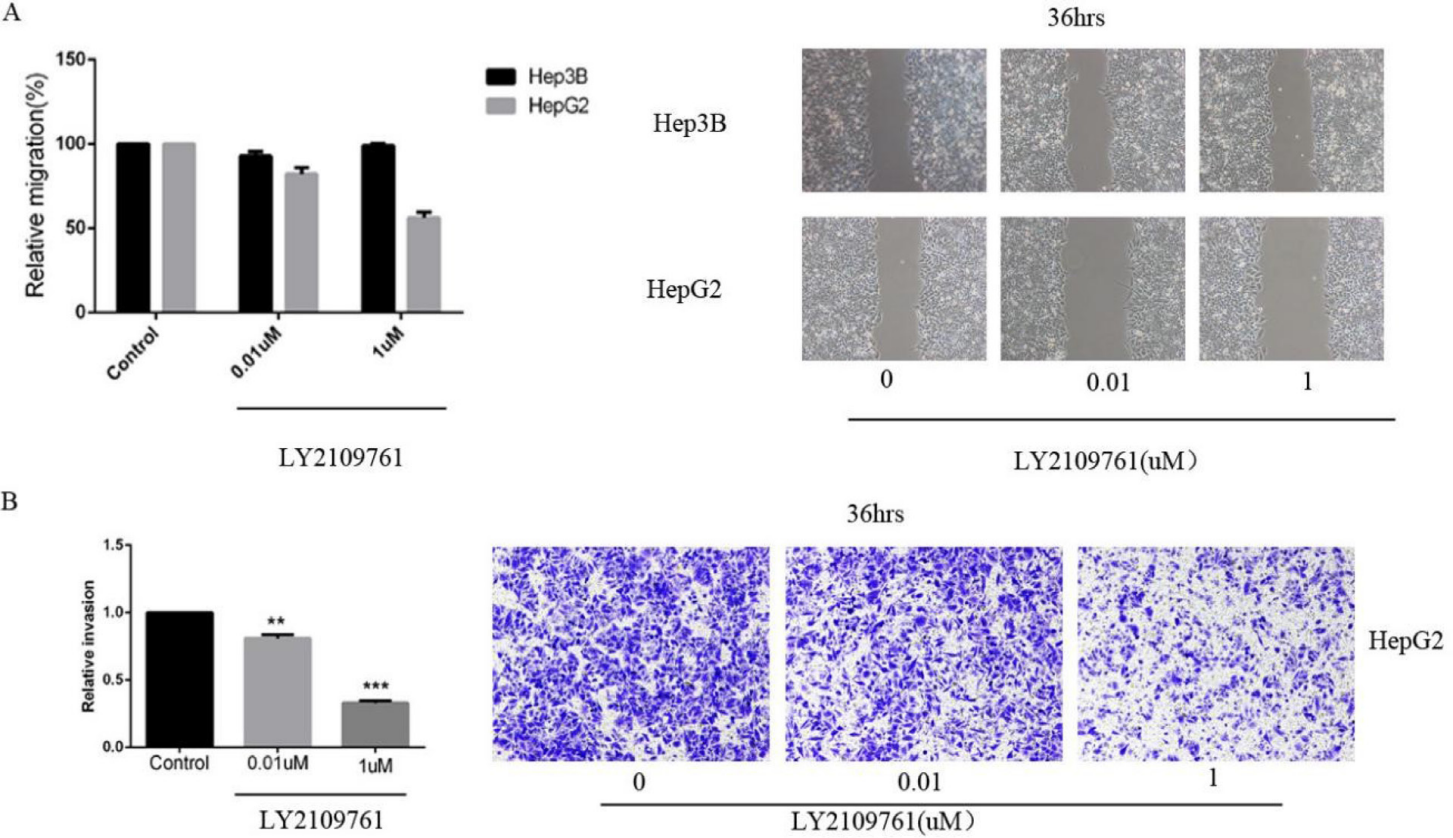
LY2109761 signifcantly suppressed both the tumor cell migration and invasion (**A**) LY2109761 could inhibit the migration of HepG2 cells at a very low dose (0.01 uM). (**B**) Consistent with the result of wound-healing assay, the invasion property of HepG2 cells was greatly suppressed by LY2109761 at a very low dose of 0.01uM.

**Figure 6:**
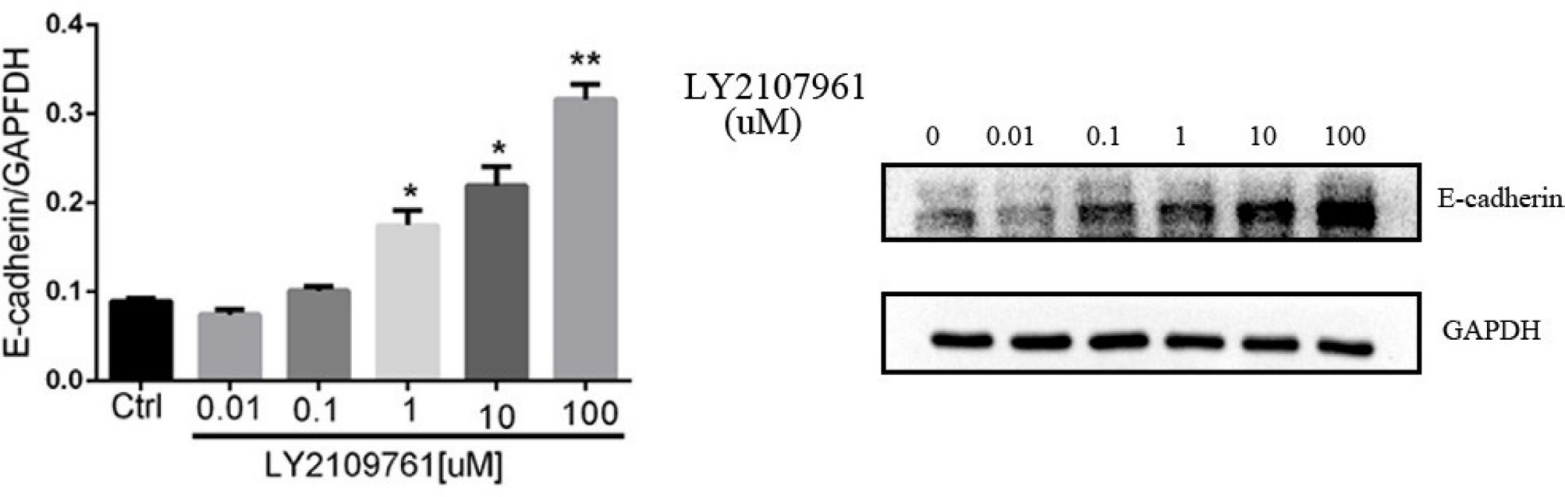
The expression of E-cadherin The migration-related protein E-cadherin was signifcantly promoted by LY2109761 in a dose dependent manner, ranging from 1 to 100 uM/L.

The inhibitory effect of LY2109761 on the migratory and invasive properties of HCC cells were investigated both *in vivo* and *in vitro*. To evaluate the alteration of tumor cell migratory and invasive properties, wound-healing and transwell-based migration assays were performed *in vitro*. As anticipated, LY2109761 signifcantly suppressed both the tumor cell migration (Figure 5A) and invasion (Figure 5B). These findings can be explained by up-regulation via a transcriptional mechanism of E-cadherin localized at the cellular membrane, where it exerted an adhesive function. And to gain better insight into the molecular mechanism underlying these biological effects, we further detected the migration-related protein E-cadherin. The data indicated that LY2109761 signifcantly promoted the expression of E-cadherin in a dose dependent manner, ranging from 1 to 100 uM (Figure 6). These results suggested that the migration and invasion of HCC cells could be dramatically blocked by LY2019761.

**Figure 7:**
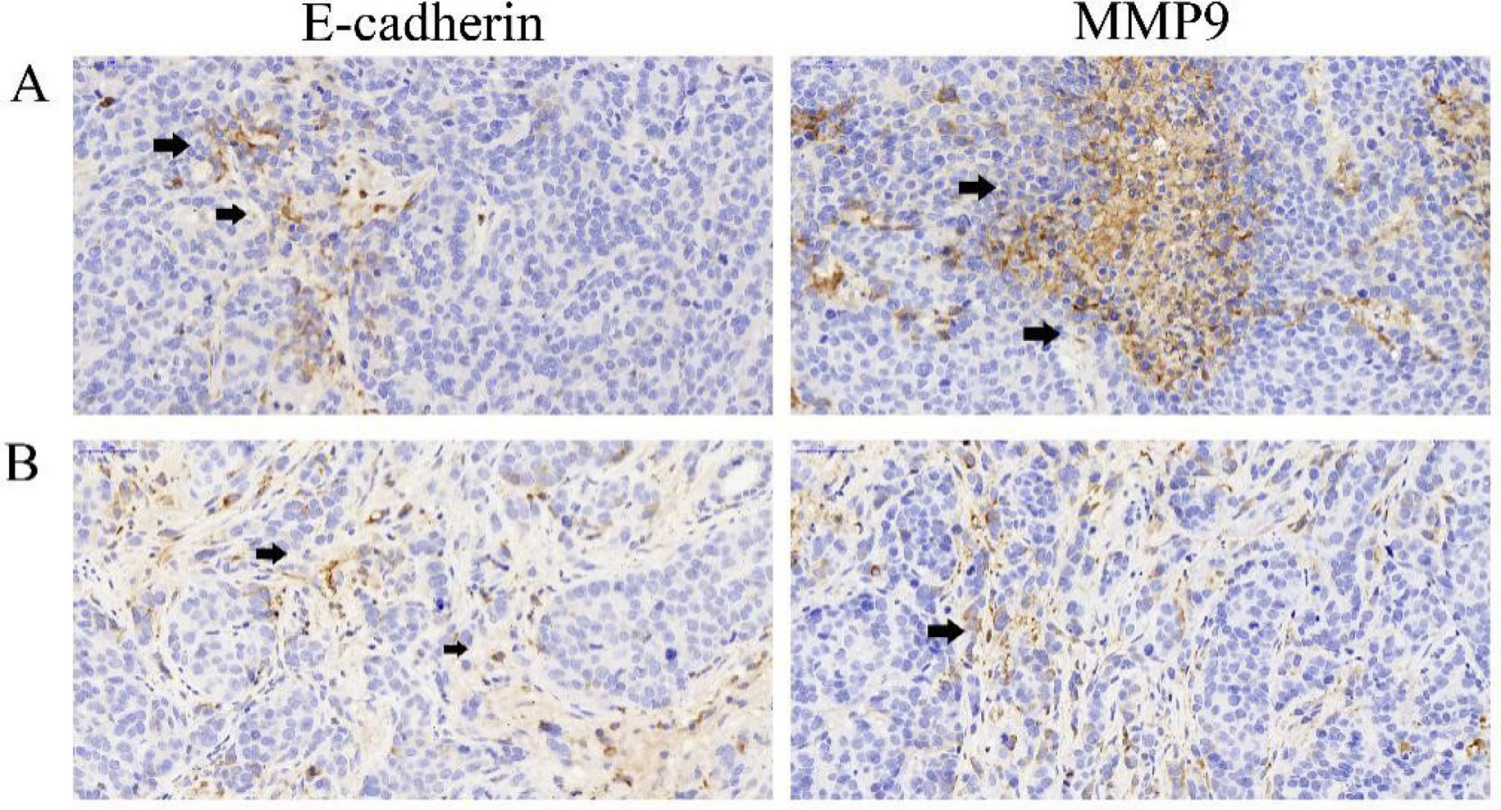
The expression of E-cadherin and MMP9 (**A**) the expression of E-cadherin(black arrow) and MMP9 (black arrow) in Group NS indicated that an overexpression of MMP9 was exsited in the tumor microenvironment of Hepatocarcinoma cells which implicated a metastatic property of the tumor cells; (**B**) the expression of E-cadherin and MMP9 in Group LY indicated that LY2109761 may play a role in down-regulating the expression of MMP9 while promoting the expression of E-cadherin.

To verify such effect of LY2109761 on inhibiting cell migration and invasion, we further evaluated the alteration of such malignant features of HCC cells in a rabbit VX2 liver cancer model. The specimens of tumor and surrounding tissues of the experimental rabbits in Group LY and Group NS were taken 3 days after TACE treatment and the expression of E-cadherin and MMP9 were also evaluated by means of the immunohistochemistry staining. And finally, from the microscopic images, we found that the expression of E-cadherin and MMP9 in Group NS (Figure 7A) were featuring a low expression of E-cadherin and an overexpression of MMP, which indicated strong migratory and invasive properties of HCC cells. And the expression of E-cadherin was upregulated whereas the expression of MMP9 were downregulated in Group LY (Figure 7B) compared with Group NS (Figure 7A), suggesting that LY2109761 may play an inhibitory role in the migratory and invasive properties of tumor cells. And those experimental results were consistent with the results obtained in the vitro model.

### LY2109761 may play a modulating role in the process of T cell transformation (*in vivo*)

**Figure 8:**
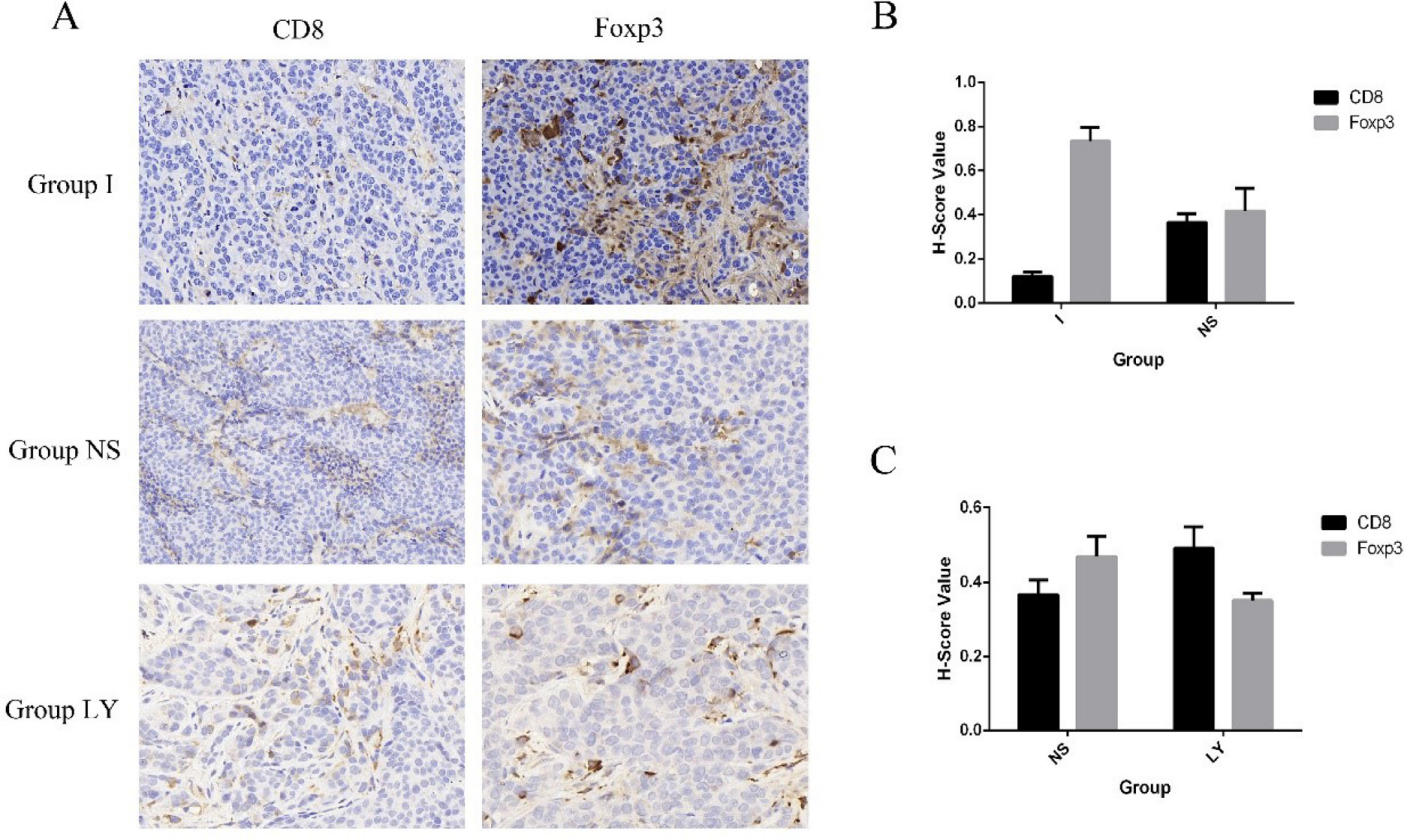
The expression of CD8 and Foxp3 (**A**) The expression of CD8 and Foxp3 in three groups, namely Group I, Group NS, Group LY. (**B** and **C**) Compared with Group NS, the expression of Foxp3 was significantly upregulated in Group I (*p* = 0.011 < 0.05), while the expression of CD8 was downregulated in Group I (*p* = 0.001 < 0.01). the expression of Foxp3 (*p* = 0.025 < 0.05) was downregulated in Group LY while the expression of CD8 (*p* = 0.042 < 0.05) was upregulated compared with Group NS.

Recently, modulating the immune response to improve it by targeting the cellular and molecular effectors involved in the pathogenesis of HCC has been a new research spotlight in the treatment of HCC. Some literatures have shown that T-lymphocytes, both CD4+ T helper cells, and CD8+ cytotoxic T-cells are significant players in immune response and are effective in inhibiting and killing tumor cells [39–41]. And previous studies have discussed the promoting role of Tregs in the pathogenesis of HCC [39]. The interest protein of Foxp3 was specifically expressed in Treg cells, whereas CD8 were specifically expressed in CTL namely CD8+ cytotoxic T-cells. Recent work has also shown that TGF-β mediates the immunosuppressive differentiation of T cells [42]. Treatment of naive T cells with TGF-β induces the expression of the transcription factor forkhead box protein P3 (FOXP3), which drives the phenotypical conversion of a naive T cell to a TReg cell [43, 44]. Based on such presumptions, in this research, we further evaluated the expression of CD8 and Foxp3 by immunohistochemistry staining in the rabbit model to gain insight into the behavior of T cell transformation before and after TACE and to further evaluate whether LY2109761 plays a role in this process. The tumor specimens of the experimental rabbits were harvested 3days after TACE treatment. And the expression of CD8 and MMP9 were analyzed and quantified by the H-score method. And to determine the condition of T cell transformation before and after TACE treatment, the statistical analysis was performed to evaluate the expression of these two factors in Group I and Group NS. The data revealed that compared with Group NS, the expression of Foxp3 was significantly upregulated in Group I (*p* = 0.011), while the expression of CD8 was downregulated in Group I (*p* = 0.001) (Figure 8A and 8B). Such observations suggested that after TACE treatment, the negative feed-back response and incomplete embolization condition of the tumor environment caused by TACE treatment stimulated the process of T cell transformation. We further compared the expression of these two factors of Group LY and Group NS to evaluate whether LY2109761 plays a role in regulating the process of T cell transformation. The data also showed an intriguing result that the expression of Foxp3 (*p* = 0.025) was downregulated in Group LY while the expression of CD8 (*p* = 0.042) was upregulated compared with Group NS (Figure 8C). In this regard, we concerned that LY2109761 may plays a role in the formation of CD8+ cytotoxic T-cells and thus participated in regulating the crosstalk between tumor cells and the tumor stoma. However, limited by time and experiment conditions, in this present study, we failed to make an intensive insight into the mechanisms of LY2109761 on its effect in regulating the behavior of T cell transformation.

### LY2109761 may play a role in inhibiting the tube formation of HUVECs

**Figure 9:**
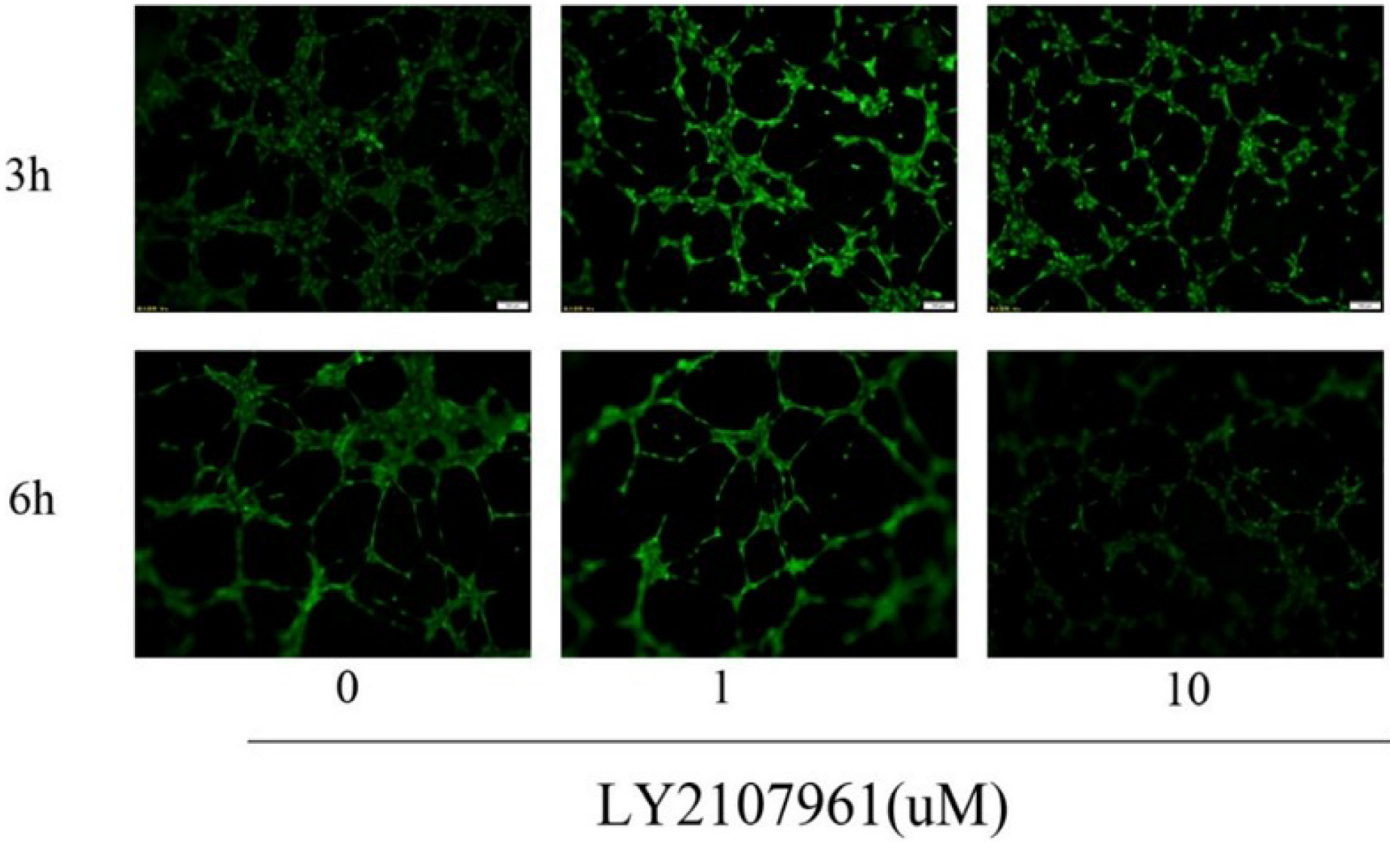
LY2109761 didn't inhibit the tube formation of HUVECs

Tumor angiogenesis is tightly correlated with the growth and metastasis of tumor cells. Some research reported that increased expression of TGF-β in tumor microenvironment may plays an important role in the pathways of tumor angiogenesis [23]. Early study have shown that TGF-β1 promotes vascular invasion by changing the functional status of integrin α5β1, and thus providing a functional explanation for HCC tumor progression [23]. Thus, we considered that LY2109761 as a TGF-β receptor inhibitor may in turn paly its inhibitory role in the process of tumor angiogenesis. And to confirm such assumption, a matrigel-based tube formation assay was conducted. And the results showed that compared with the control group (LY, 0 uM /L), a significant decrescence of vascular tube formation wasn’t observed when the concentration of LY 2109761 increaesd( from 0 uM /L to 10 uM /L) the incubation time of 3hours and 6hours (Figure 9). So we considered that LY2109761 may not have an inhibitory effect of tube formation of HUVECs.

## MATERIALS AND METHODS

### In Vitro

#### Cell cultures, antibodies and reagents

The human HCC cell lines HepG2, Hep3B and Human Umbilical Vein Endothelial Cells (HUVEC) were obtained and cultured in Dulbecco’s Modified Eagle’s Medium (DMEM, Gibco BRL, supplemented with 10% foetal bovine serum, 100 mg/ml penicillin, and 100 mg/ml streptomycin. The cells were incubated at 37°C in a humidified atmosphere at 5% CO_2_. The TGF-b receptor kinase inhibitor LY2109761 was purchased from Eli Lilly (Indianapolis, IN). Cell Counting kit-8 was purchased from Sigma (Milan, Italy), monoclonal blocking antibody against human E-cadherin, SHE78–7, from Alexis (Lausanne, Switzerland) and polyclonal antibodies against phospho-Smads were purchased from Cell Signaling Technology Inc. (Danvers, MA) and monoclonal antibody against β-actin were purchased from Sigma, while growth factor–reduced (GFR) Matrigel were purchased from BD.

### Cytotoxicity assays

The cytotoxicity of LY2109761 was determined by the Cell Counting Kit-8 assay. The HCC cell line HepG2 Cells were plated and cultured for 24 hours in a humidified atmosphere at 5% CO2, 37°C, and then supplemented with LY2109761 at the following concentrations: 2, 4, 8, 16, and 32 uM as well as the CCK8 solution(10 ul). Each experimental condition was reproduced in 8 wells, and each experiment would be repeated for 3 times. To confirm the cytotoxic data, cells were incubated under the described conditions for 1–4 hours and the absorbance was measured at 450 nm using the microplate reader.

### Wound-healing assay

The HCC cell lines HepG2, Hep3B were seeded in a 6-well plate to form a confluent monolayer in complete medium. The monolayer was pretreated with LY2109761 at the following concentrations: 0, 0.01uM and 1uM for 36hrs before being scratched by a plastic tip. And then the wound closures were monitored under microscopy at × 100 magnifcation.

### Migration and invasion assay

To evaluate the alteration of tumor cell migratory and invasive properties, cell migration assays were performed. Cells were serum-starved overnight and then cultured in the absence or presence of LY2109761 at concentrations ranging from 0.01 uM to 1 uM, and the time was ranging from 0 hour to 36 hours.

### Matrigel-based tube formation assay

The co-cultures on matrigel system was used to assess the inhibitory effect of LY2109761 on the angiogenic capability of HepG2 and Hep3B cells. Briefly, HepG2 and Hep3B cells were embeded in 100 µL liquid matrigel and seeded into 96-well plates. After overnight incubation, 5 × 103 Human Umbilical Vein Endothelial Cells (HUVECs) in 100 µL of the complete medium were introduced onto the top of the solidified HepG2 and Hep3B matrigel suspension with LY2109761 of different concentrations. The co-cultures on matrigel were inspected and photographed at 3 hrs and 6hrs, respectively, following the overlaid of HUVECs under an inverted light microscope at 200 × magnification. Five independent fields were detected for each well and the average number of tubes/field was calculated.

### Western blot analysis

The cells were resuspended in cell lysis buffer (pH 8.0) that contained 50 mM Tris–HCl, 150 mM NaCl, 5 mM EDTA, 1% NP40, 0.05% PMSF, 2 mg/ml aprotinin, and 2 mg/ml leupeptin. Approximately 30 mg of protein/well was loaded onto the gels. The targeted protein levels were determined using western blotting, as described previously.

### Statistical analysis

All the data were presented as mean ± SEM. Each value is the mean of at least three separate experiments in each group. Student’s *t*-test was conducted using GraphPad Prism software. *P* < 0.05 was considered significant.

### Animal experiment (*In Vivo*)

#### Animals of experiment

New Zealand white rabbits weighing 2.5–3.0 kg were purchased from the Experimental Animal Center of Huazhong University of Science and Technology for experimental use of these studies. All the experiment protocol were approved by the Animal Experiment Committee of Institute for Huazhong University of Science and Technology.

The rabbit VX2 hepatocellular carcinoma model was established as follows: (1) The VX2 tumor cells were implanted on the inside of a hind leg muscles of rabbits, 2 weeks after the implantation substantial mass were palpable, that is tumor-bearing rabbits. (2) One tumor-bearing rabbit was sacrificed and the auxetic VX2 tumor was separated under sterile conditions and is made into 1 × 1 × 1 mm3 pieces of tumor tissue. Then the pieces were stored in physiological saline. (3) The rabbit was operated in the abdominal median incision and subsequently, VX2 tumor was inoculated into the left lobe of the liver in the candidate hepatic area and the puncture was covered by gelatin and then the left lobe of the liver was replaced into the abdomen. (4) All of the rabbits were given intramuscular injection of penicillin for three days after the implantation of VX2 tumors. The tumors were allowed to grow in the rabbits’ livers for 14 days.

### Measurement of tumor size before TACE procedure

The perfusion CT was used to estimate the tumor size of the experiment rabbits which implanted with VX2 tumor tissues 14 days later. All the rabbits were anaesthetized by the chloral hydrate(10%). After scanning, all the images acquired were processed by the Syngo Fastview image processing system, and the size, location, shape and the presence of necrosis and intrahepatic metastasis of the implanted tumor were analyzed by twos senior doctors of radiology department. The maximum diameter (A1) and transverse diameter (B1) of tumor were separately recorded and the tumor size (V1) was calculated as “V_1_ = A_1_×B_1_^2^/2 ”.

### TACE procedure

TACE treatment were performed on those rabbits which implanted with VX2 liver tumor one day after they were scanned with perfusion CT. 40 rabbits were randomly divided into four groups, which were respectively named as Group I-LY, Group I, Group LY, Group NS, and each group included 10 rabbits: Group I-LY(3mL ultra liquid iodized oil containing 50 mg LY2109761-DMSO suspension, TACE), Group I (3mL ultra liquid iodized oil supplemented with gelatin sponge, TACE), Group LY (the mixture of 3 mL normal saline solution with 50mg LY2109761-DMSO suspension), Group NS(3mL normal saline solution).

All rabbits were treated under general anesthesia, and then the arteria cruralis of each rabbit was dissected bluntly. Subsequently, a catheter guide wire was moved to the hepatic tumor feeding artery from arteria cruralis under the guidance of DSA. Then different drugs of different groups were separately injected into the feeding artery of those rabbits. Finally, the arteria cruralis of each rabbit was sutured and all of the rabbits were given intramuscular injection of penicillin for three days.

### Measurement of tumor size after TACE procedure

All the experimental rabbits which under TACE treatment were scanned by perfusion CT 7 days after the operation. The size, location, shape and the presence of necrosis and intrahepatic metastasis of the rabbits were reanalyzed. The maximum diameter (A2) and transverse diameter (B2) of tumor were separately recorded and the tumor size (V2) was calculated as “V_2_ = A_2_×B_2_^2^/2. The tumor growth rate was calculated as well.

### Tissue analysis

All animals were euthanized with an overdose of chloral hydrate (10%) (Laboratory Animal Center, HuaZhong University of Science and Technology). The tumor and the surrounding liver tissues were removed. The tissue sample was bisected and each half included tumor and the surrounding liver tissues. All the liver tissues were embedded in paraffin and serially cut for immunohistochemisry analysis. Immunohistochemistry staining was performed on formal in fixed paraffin-embedded tumor tissue sections. The sections were deparaffnized, rehydrated and stained with primary antibodies over night at 4°C. These antibodies were detected with biotinylated secondary antibody, followed by incubation with horseradish peroxidaseconjugated streptavidin-biotin complex. Finally, the sections were developed in diaminobenzidine and visualized under a light microscope.

### Statistical analysis

All data were presented as mean ± SD. The measurement data was analyzed statistically by One-Way ANOVA and double-factor variance analysis. P ＜ 0.05 was considered as statistically significant difference. All the Figures were drawn by Graphpad prism V6.0.

## CONCLUSIONS

In the present study, we for the first time combined TACE therapy with the TGF-β kinase receptor inhibitor--- LY2109761 for the treatment of liver cancer. We have evaluated the inhibitory effect of LY2109761 on modulating tumor cell proliferation and migration properties in a vitro model as well as evaluated the therapeutic effect of it on inhibiting tumor growth after TACE in a rabbit VX2 liver cancer model. Based on those observations described above, we concluded that LY2109761 not only inhibits cell proliferation in a vitro model but also inhibits tumor growth while increase tumor necrosis in the rabbit liver cancer model. And by western blot assay, we concluded that down-regulation phosphorylation of Smad-2 in the TGF/Smad pathway which induced by LY2109761 may contributed to such inhibitory effect of this agent. And to gain a comprehensive insight into the effect of LY2107961, we have further evaluated the inhibitory effect of it on cell migration and invasion both *in vivo* and *in vitro*. And our data have shown that LY2109761 can also block the migration and invasion of HCC cells by decreasing the expression of migration related protein E-cadherin whereas promting the expression of invasion related protein MMP9. Furthermore, in this present study, we have shown that LY2109761 may plays a role in the formation of CD8+ cytotoxic T-cells and thus participated in regulating the crosstalk between tumor cells and the tumor stoma. However, the mechanisms underlying such behaviors is not yet clear by now and needs to be further evaluated.

In brief, our study has for the first time confirmed that combing LY2109761 with TACE on the treatment for VX2 rabbit liver cancer can help inhibit tumor growth as well as increase the tumor cell necrosis after TACE. Our findings may provide new treatment options and stragies for the treatment of liver cancer.

## Abbreviations

TACE: Transcatheter Arterial Chemoembolization
HCC: Hepatocellular Carcinoma
BCLC: Barcelona Clinic Liver Cancer
TGF: Transforming Growth Factor
MTAs: Molecular Targeted Agents
ADRs: AdverseRreactions
HUVECs: Human Umbilical Vein Endothelial Cells
TGR: Tumor Growth Rate
